# Can Obesity Serve as a Barrier to Minimally Invasive Mitral Valve Surgery? Overcoming the Limitations—A Multivariate Logistic Regression Analysis

**DOI:** 10.3390/jcm13216355

**Published:** 2024-10-24

**Authors:** Sadeq Ali-Hasan-Al-Saegh, Florian Helms, Khalil Aburahma, Sho Takemoto, Nunzio Davide De Manna, Lukman Amanov, Fabio Ius, Jan Karsten, Alina Zubarevich, Bastian Schmack, Tim Kaufeld, Aron-Frederik Popov, Arjang Ruhparwar, Jawad Salman, Alexander Weymann

**Affiliations:** 1 Department of Cardiothoracic, Transplantation and Vascular Surgery, Hannover Medical School, 30625 Hannover, Germanyius.fabio@mh-hannover.de (F.I.);; 2 Center for Transplantation Sciences, Department of Surgery, Massachusetts General Hospital and Harvard Medical School, Boston, MA 02114, USA; 3 Department of Anaesthesiology and Intensive Care Medicine, Hannover Medical School, 30625 Hannover, Germany; karsten.jan@mh-hannover.de

**Keywords:** minimally invasive mitral valve surgery, mitral valve replacement, mitral valve repair, obesity, BMI

## Abstract

**Background/Objectives:** Over the past two decades, significant advancements in mitral valve surgery have focused on minimally invasive techniques. Some surgeons consider obesity as a relative contraindication for minimally invasive mitral valve surgery (MIMVS). The aim of this study is to evaluate whether the specific characteristics of obese patients contribute to increased surgical complexity and whether this, in turn, leads to worse clinical outcomes compared to non-obese patients. Furthermore, we aim to explore whether these findings could substantiate the consideration of limiting this treatment option for obese patients. We investigated the outcomes of MIMVS in obese and non-obese patients at a high-volume center in Germany staffed by an experienced surgical team well-versed in perioperative management. **Methods:** A total of 934 MIMVS were performed in our high-volume center in Germany from 2011 to 2023. Of these, 196 patients had a BMI of 30 or higher (obese group), while 738 patients had a BMI below 30 (non-obese group), all of whom underwent MIMVS by right minithoracotomy. Demographic information, echocardiographic assessments, surgical data, and clinical outcome parameters were collected for all patients. **Results:** There was no significant difference in in-hospital, 30-day, and late mortality between groups (obese vs. non-obese: 6 [3.0%] vs. 14 [1.8%], *p* = 0.40; 6 [3.0%] vs. 14 [1.8%], *p* = 0.40; 13 [6.6%] vs. 39 [5.3%], *p* = 0.48, respectively). Respiratory insufficiency and arrhythmia occurred more frequently in the obese group (obese vs. non-obese: 25 [12.7%] vs. 35 [4.7%], *p* < 0.001; 35 [17.8%] vs. 77 [10.4%], *p* = 0.006). **Conclusions:** Obesity was not associated with increased early or late mortality in patients undergoing MIMVS. However, obese patients experienced higher incidences of postoperative complications, including respiratory insufficiency, arrhythmias, delirium, and wound dehiscence. Nonetheless, a multivariate logistic regression analysis indicated that obesity itself does not contraindicate MIMVS and should not be viewed as a barrier to offering this minimally invasive approach to obese patients.

## 1. Introduction

Obesity has become a significant global health concern, with its prevalence continuing to rise worldwide. The global prevalence of obesity has tripled in recent decades, with around 40% of men classified as overweight (body mass index [BMI) of 25–30 kg/m^2^) and 13% classified as obese (BMI > 30 kg/m^2^) in 2016 [1]. Obesity is recognized as a considerable risk factor for various health conditions, including diabetes, cardiovascular disorders, and respiratory complications [2]. Given the link between obesity and multiple cardiovascular risk factors, it is likely that the number of cardiac surgery patients with obesity will increase significantly in the coming years and decades. In the context of cardiac surgery, obesity is associated with an increased likelihood of complications such as acute kidney injury and impaired wound healing, longer hospital stays, and the need for re-exploration after conventional cardiac surgeries [3,4,5]. The absolute prevalence of primary mitral valve regurgitation, one of the most common degenerative heart valve diseases, has significantly increased, with a 70% rise observed from 1990 to 2017 [6]. These rising trends necessitate a deeper understanding of how obesity impacts the outcomes of cardiac surgery, particularly in patients with mitral valve disease.

Over the past two decades, significant advancements in mitral valve surgery have focused on minimally invasive techniques, including minithoracotomy and robotic- and video-assisted methods. Compared to a conventional full sternotomy, minimally invasive techniques offer improved short- and mid-term outcomes, including a reduced severity of pain after surgery, shorter hospitalization, and faster recovery [7]. However, several challenges remain, particularly regarding the suitability of minimally invasive mitral valve surgery (MIMVS) for obese patients. Some surgeons consider obesity a relative contraindication for MIMVS based on several factors, such as a limited access to the mediastinum, reduced visibility associated with the increased abdominal weight elevating the right hemidiaphragm in a supine position, a higher ventilation pressure demand and risk of barotrauma, and difficulties in the management of venous drainage and arterial line pressures [8,9,10]. Despite these challenges, minimally invasive approaches may offer an attractive alternative for obese patients, potentially reducing the risk of complications associated with a sternotomy without compromising safety and efficacy.

The aim of this study is to evaluate whether the specific characteristics of obese patients contribute to increased surgical complexity and whether this, in turn, leads to worse clinical outcomes compared to non-obese patients. Furthermore, we aim to explore whether these findings could substantiate the consideration of limiting this treatment option for obese patients. We investigated the outcomes of MIMVS in obese and non-obese patients at a high-volume center in Germany staffed by an experienced surgical team well-versed in perioperative management. The novelty of our study, in comparison to previous studies, lies in several key aspects. First, we utilized a larger sample size of patients who underwent MIMVS through minithoracotomy, which enhances the reliability of our findings. Additionally, we conducted comprehensive follow-ups for each patient until 2024 to assess late mortality rates, providing valuable insights into long-term outcomes. Furthermore, we employed specific statistical methods, including a multivariate regression analysis, to investigate the exact impact of obesity on clinical outcomes and complications following MIMVS.

## 2. Materials and Methods

### 2.1. Study Population

From 2011 to 2023, a total of 934 MIMVS were performed in our high-volume center in Germany. Of these, 196 patients had a BMI of 30 or higher (obese group), while 738 patients had a BMI below 30 (non-obese group), all of whom underwent MIMVS by right minithoracotomy. We collected perioperative and postoperative (post-op) data from our database. The inclusion criteria encompassed all etiologies of mitral valve disease, including degenerative, ischemic, rheumatic, and infective causes. Patients with severe extracardiac arteriopathy that precluded the establishment of a cardiopulmonary bypass (CPB) through the groin vessels, as well as those with a significantly impaired left ventricular ejection fraction, were excluded from the study.

### 2.2. Physical Measurements

Height was measured without shoes using a standard wall-mounted stadiometer, and weight was recorded using calibrated physician-grade scales. Trained personnel conducted these measurements to ensure accuracy. Body mass index (BMI) was calculated using the standard formula: BMI = weight (kg)/height (m)^2^.

### 2.3. Ethical Statement

In accordance with local German protocols, study approval by the institutional ethical review board was waived given the retrospective and non-interventional design of this study.

### 2.4. Surgical Technique

The surgical techniques, including the right minithoracotomy approach, as well as the perfusion strategies and aortic clamping techniques employed in this study, have been previously described by our team [11]. All surgeries were conducted through a right minithoracotomy, with continuous carbon dioxide insufflation maintained throughout the procedure. In brief, a cardiopulmonary bypass (CPB) was typically initiated via the right femoral vessels, and single-lung ventilation was utilized. Initially, a two-stage venous cannula was placed into the superior vena cava using echocardiographic guidance, followed by the insertion of an arterial cannula. The pericardium was incised 3 to 4 cm above the phrenic nerve. Unfractionated heparin was administered intravenously to achieve an activated clotting time exceeding 450 s, which was fully reversed with protamine after removal of the venous cannula.

### 2.5. Follow-Up and Patient Data Collection

All patients were followed up for 30 days after surgery, and their survival was tracked until August 2024. Demographic information, echocardiographic assessments, surgical data, and clinical outcome parameters were collected for all patients. This included the incidence of post-op complications such as wound dehiscence, arrhythmia, right ventricular failure, new-onset atrial fibrillation (NOAF), new myocardial infarction, the need for pacemaker implantation, thromboembolic events, respiratory insufficiency, the necessity for cardiopulmonary resuscitation, acute renal failure requiring dialysis, ischemic stroke, delirium, intracranial hemorrhage, major bleeding necessitating re-thoracotomy, and sepsis. Additional metrics included intubation duration, duration of catecholamine therapy, and transfusion of blood cells or products. Post-op early mortality was defined as death occurring during the hospital stay or within the first 30 days after surgery, while late mortality referred to deaths occurring beyond 30 days.

### 2.6. Echocardiographic Assessment

Transthoracic echocardiography was routinely performed both before surgery and prior to discharge. The echocardiographic parameters collected included left ventricular ejection fraction (LVEF), grades of mitral valve insufficiency (MI) II, III, and IV, and grades of mitral valve stenosis (MS) II and III.

### 2.7. Statistical Analysis

Data analysis was performed using SPSS version 28.01.1. Continuous variables were summarized as the mean with standard deviation (SD), while categorical variables were reported as counts and percentages relative to the total sample size. A Mann–Whitney U test was utilized to compare continuous variables, whereas a Chi-square test was used for categorical comparisons. A Fisher’s exact test was conducted when any cell in the cross-tabulation had an expected count of less than 5. Multivariate logistic regression models were fitted for each dichotomous outcome, while multivariate linear regression models were used for continuous outcome variables. A multivariate logistic regression analysis was selected for this study as it facilitates the prediction of a binary outcome, such as the occurrence or non-occurrence of postoperative complications, by considering multiple independent variables, including obesity and various patient characteristics. Results from the regression analysis were reported as odds ratios (OR) along with 95% confidence intervals (CIs). Each regression model was conducted independently. A *p*-value of less than 0.05 was considered statistically significant.

## 3. Results

### 3.1. Patient Demographics and Perioperative and Procedure Characteristics

**Figure 1:**
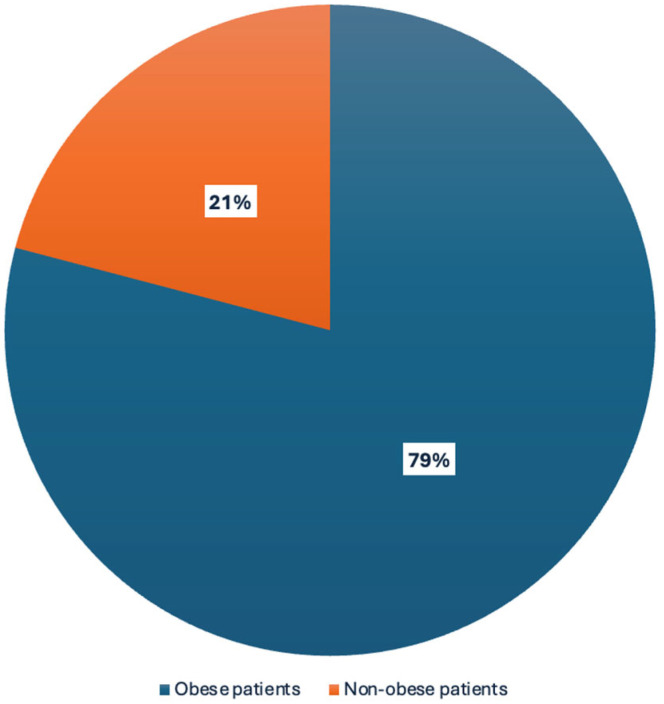
Distribution of patients according to obesity (BMI ≥ 30) and non-obesity (BMI < 30).

Of the 934 patients who underwent MIMVS, 196 patients (21.0%) had a BMI ≥ 30 kg/m^2^, and 738 patients (79.0%) had a BMI < 30 kg/m^2^ (Figure 1). Patient demographics and pre-and intraoperative data are summarized in Table 1 and Table 2. Male gender (obese: 99 [50.5%] vs. non-obese: 411 [55.6%], *p* = 0.02), insulin-dependent diabetes mellitus (obese: 18 [9.1%] vs. non-obese: 18 [2.4%], *p* < 0.001), pulmonary hypertension (obese: 104 [53.0%] vs. non-obese: 309 [41.8%], *p* = 0.03), arterial hypertension (obese: 167 [85.2%] vs. non-obese: 460 [62.3%], *p* < 0.001), hyperlipidemia (obese: 118 [60.2%] vs. non-obese: 304 [22.4%], *p* < 0.001), atrial fibrillation (obese: 111 [56.6%] vs. non-obese: 313 [42.34], *p* < 0.001), mitral valve stenosis (obese: 23 [11.7%] vs. non-obese: 34 [4.6%], *p* < 0.001), mitral valve prolapse (obese: 83 [42.3%] vs. non-obese: 461 [62.4%], *p* < 0.001), endocarditis (obese: 8 [4.0%] vs. non-obese: 67 [9.0%], *p* = 0.02), and maximum mitral valve gradient (obese: 10.1 ± 5.2 vs. non-obese: 8.9 ± 5.1 mmHg, *p* = 0.007) were significantly different between groups. Mechanical mitral valve replacement was performed more frequently in the obese group (obese: 35 [17.8%] vs. non-obese: 73 [9.8%], *p* = 0.004), and mitral valve repair was performed more frequently in the non-obese group (obese: 102 [52.0%] vs. non-obese: 479 [64.9%], *p* < 0.001). Among mitral valve repair, neochordae was utilized more frequently in the non-obese group (obese: 61 [31.1%] vs. non-obese: 343 [46.4%], *p* < 0.001). The obese group needed a significantly longer duration of surgery (obese: 224.1 ± 65.7 vs. non-obese: 210.8 ± 54.5 [46.4%], *p* < 0.001). All other demographics were not significantly different between groups.

### 3.2. Postoperative Outcomes and Echocardiographic Data

**Figure 2:**
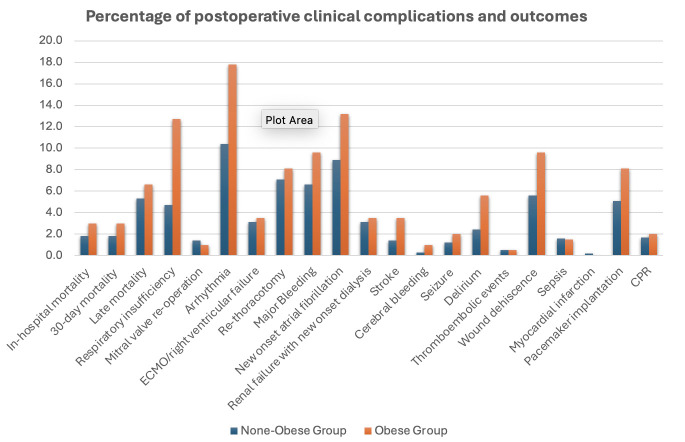
Percentage of postoperative clinical complications and outcomes.

Post-op outcomes are shown in Table 3. There was no significant difference in in-hospital, 30-day, and late mortality between groups (obese vs. non-obese: 6 [3.0%] vs. 14 [1.8%], *p* = 0.40; 6 [3.0%] vs. 14 [1.8%], *p* = 0.40; 13 [6.6%] vs. 39 [5.3%], *p* = 0.48, respectively). Respiratory insufficiency and arrhythmia occurred more frequently in the obese group (obese vs. non-obese: 25 [12.7%] vs. non-obese: 35 [4.7%], *p* < 0.001; 35 [17.8%] vs. non-obese: 77 [10.4%], *p* = 0.006), and the incidence of new-onset atrial fibrillation (NOAF), stroke, and wound dehiscence were near significant in two groups (obese vs. non-obese: 26 [13.2%] vs. 66 [8.9%], *p* = 0.07; 7 [3.5%] vs. 11 [1.4%], *p* = 0.07; 19 [9.6%] vs. 42 [5.6%], *p* = 0.06, respectively). The incidence of delirium was higher in the obese group (obese: 11 [5.6%] vs. non-obese: 18 [2.4%], *p* = 0.003) (Figure 2). There was no significant difference in other variables.

Echocardiographic outcomes are displayed in Table 4. Although the non-obese group showed significantly higher preoperative (pre-op) LVEF, post-op LVEF was comparable among groups (obese vs. non-obese: 50.7 ± 18.6 vs. 55.0 ±16.3%, *p* = 0.002; 51.1 ± 13.3 vs. 52.0 ± 11.2%, *p* = 0.37). Grade II mitral stenosis (MS) was observed more frequently in the obese group preoperatively (obese: 8 [4.0%] vs. non-obese: 8 [1.0%], *p* = 0.007). Post-op echocardiography revealed a near-significant higher incidence of grade I MI in the non-obese group (obese: 27 [13.7%] vs. non-obese: 148 [20.0%], *p* = 0.07), while grade II or higher MI and MS were rarely observed in both groups.

### 3.3. Regression Model: Impact of Obesity on Mortality and Postoperative Outcomes

- *Mortality*

Obesity was not significant for in-hospital and 30-day mortality in the multivariate logistic regression analysis (odds ratio (OR): 3.82, 95% CI: [0.15–92.27], *p* = 0.4; OR: 1.61, 95% CI: [0.12–20.36], *p* = 0.71, respectively) (Table 5). No other pre-op conditions or procedural characteristics were not a risk factor for mortality.

- *Arrhythmia*

Obesity was not an independent predictive factor for post-op arrhythmia (OR: 1.18, 95% CI: [0.68–2.06], *p* = 0.54). Both mechanical MV replacement and MV repair were protective factors (OR: 0.23, 95% CI: [0.08–0.64], *p* < 0.001; OR: 0.47, 95% CI: [0.26–0.85], *p* = 0.01, respectively). Pre-op AF was a substantial risk of post-op arrhythmia (OR: 3.23, 95% CI: [1.91–5.48], *p* < 0.001).

- *Respiratory insufficiency*

Obesity did not increase the risk of post-op respiratory insufficiency (OR 0.96, 95% CI 0.44–2.10, *p* = 0.93). Pre-op LVEF was associated with the risk of post-op respiratory failure (OR 0.97, 95% CI 0.96–0.99, *p* = 0.01), whereas pre-op pulmonary hypertension showed borderline significance as a predictive factor (OR 2.01, 95% CI 0.98–4.09, *p* = 0.05).

- *Wound dehiscence and delirium*

Our logistic regression analysis revealed obesity was not a risk factor for either wound dehiscence or delirium (OR: 1.71, 95% CI: [0.885–3.336], *p* = 0.11; OR: 1.03, 95% CI: [0.34–3.13], *p* = 0.95, respectively). Furthermore, no significant independent predictive factors were identified for either wound dehiscence or delirium.

## 4. Discussion

Our study revealed that obesity did not contribute to an increased risk of either early or late mortality for patients who underwent MIMVS. Although respiratory insufficiency, arrhythmia, delirium, and wound dehiscence were observed more frequently in the obese group, obesity was not identified as an independent factor for these postoperative morbidities. Notably, despite both groups having a comparable mean patient age, mechanical MV replacement was more frequently performed in the obese group. In contrast, MV repair was more common in the non-obese group. Additionally, the obese group had significantly longer operative times, and a higher incidence of grade II MS was observed in postoperative echocardiography. While these findings suggest that the complexity of surgery may increase in obese patients, leading to a higher risk of certain complications, obesity alone does not appear to contraindicate MIMVS.

Obesity has emerged as a major global health issue, with prevalence rates continuing to rise globally, including across Europe. The World Health Organization (WHO) reported that the global obesity rate has almost doubled since 1980, and nearly 60% of adults in the European region are classified as overweight (BMI ≥ 25 kg/m^2^) or obese (BMI ≥ 30 kg/m^2^) [12]. The age group with the highest prevalence is those between 65 and 74 years old [12]. In Germany, the obesity rate stands at 19%, with similar rates observed between men and women [13].

Studies consistently have shown that obesity is associated with a higher risk of prolonged mechanical ventilation, pneumonia, deep sternal wound infections, acute renal failure, post-op AF, increased medical costs, and extended ICU/hospital stays in patients undergoing cardiac surgery [4,14,15]. While some large population studies have found no association between obesity and increased mortality [4,14] including in patients with morbid obesity (BMI ≥ 40 kg/m^2^) [14], other studies have reported a higher mortality risk in obese patients [15]. However, neither obesity nor morbid obesity has been linked to an increased mortality in patients undergoing minimally invasive cardiac surgery, including MIMVS or robotic procedures, which is consistent with our findings [8,16,17].

Mariscalco et al., using data from the UK national registry and a meta-analysis, reported that obesity (BMI 30–40) was associated with lower mortality, while underweight patients exhibited increased mortality after cardiac surgery compared to those with normal weight [17]. Even after adjusting for confounding factors in a multivariate analysis, mortality was found to be 15–20% lower in the obese population [17]. Contrary to the expectation that obese patients would be at higher risk, this phenomenon of lower mortality in obese patients has been observed in several studies and is known as the “obesity paradox” [18]. This paradox has been documented not only in cardiac surgery outcomes but also in conditions such as heart failure, chronic obstructive lung disease, cancer, and chronic kidney disease [19]. Mariscalco and colleagues further suggested that the observed reduction in mortality among obese patients might challenge the conventional practice of advising preoperative weight loss in this population [17]. However, Carnethon et al. have pointed out the limitations of observational studies, emphasizing the potential for selection bias and the challenges of establishing causal relationships in such research designs [20]. While there may be short-term benefits for obese patients undergoing cardiac surgery, the long-term impact of obesity remains uncertain. Obese patients have higher risks for deep sternal wound infections and dialysis, both of which could adversely affect long-term outcomes [17]. Minimally invasive cardiac surgery offers the advantage of avoiding sternotomy, which can mitigate the risk of deep sternal wound infections. Additionally, in our study, there was no significant difference in the incidence of dialysis-requiring renal failure between obese and non-obese patients. These results suggest that obese patients may benefit from a minimally invasive approach by potentially avoiding these serious complications, highlighting the advantages of this approach for managing obese patients undergoing cardiac surgery.

Although mortality rates between obese and non-obese patients undergoing MIMVS have been shown to be comparable, obesity has been associated with an increased risk of complications, even in minimally invasive cardiac surgery [8,21,22]. These complications include a higher incidence of arrhythmias, surgical site infections, prolonged ventilation times, and extended hospital stays. Similarly, in our analysis, the obese group had a significantly higher incidence of respiratory insufficiency, arrhythmia, delirium, and wound dehiscence. On the other hand, Kitahara et al. reported no significant differences in morbidity between obese and non-obese patients undergoing robotic coronary artery bypass grafting [16]. However, it is important to note that approximately 60% of these procedures were total endoscopic in nature, with minimal incisions, and the use of CPB was limited to around 30% [16].

The pathophysiology of obesity-related complications in cardiac surgery is multifaceted. Impaired wound healing in obese patients can be attributed to poor vascularity and the tenuous structure of adipose tissue, leading to relative vascular insufficiency and decreased oxygen tension [23]. This may result in reduced collagen synthesis, impaired infection resistance, and compromised wound healing processes [23]. These factors may explain the increased wound healing disturbances in obese patients undergoing MIMVS, despite the smaller incisions compared to median sternotomy. Obesity also increases the risk of postoperative arrhythmias, particularly atrial fibrillation, due to excess adipose tissue altering atrial electrophysiology and obesity-associated atrial dilation [24]. Additionally, obesity leads to reduced lung capacity, functional residual volume, and shallow, fast breathing, potentially explaining the higher incidence of postoperative respiratory failure [25]. Interestingly, some observational studies report an “obesity paradox” for postoperative delirium, suggesting that a higher body weight may have a protective effect [26]. However, advanced age remains a significant risk factor for postoperative delirium, even within obese patient populations [27]. In our cohort, the obese group had a statistically near-significant higher age, which may have contributed to the higher incidence of delirium. Nevertheless, the mechanisms linking obesity and postoperative delirium remain unclear, warranting further investigation.

Santana et al. reported lower morbidity and mortality, including reduced incidences of renal failure, deep wound infections, blood transfusion, and shorter ICU and hospital stays, in patients who underwent a minimally invasive approach for isolated valve lesions compared to a median sternotomy [28]. However, aortic cross-clamp and CPB times were significantly longer in the minimally invasive group, suggesting that this approach may present greater technical challenges in obese patients compared to those with a normal body weight [28]. In our study, CPB times were similar between the groups; however, the overall duration of surgery was longer in the obese group, and MV replacement was more frequently performed than MV repair. Additionally, postoperative grade II MS was observed more often in obese patients. These findings indicate that specific technical difficulties associated with obesity may influence procedural choices, but it is also important to mention that the obese group had a significantly higher proportion of patients with MS, for whom valve replacement was the only viable option. Hadaya et al. reported that despite increased procedural complexity, high-volume centers demonstrated an independent association between greater operative volume and reduced hospitalization costs and mortality following elective cardiac operations [29]. Even though obesity itself may not be an independent risk factor for increased mortality or complications, it is evident that the technical and postoperative management challenges associated with obesity require special considerations. Therefore, such procedures should be concentrated at experienced high-volume centers where these challenges can be more effectively managed.

The clinical implications of our findings are noteworthy for the management of obese patients undergoing MIMVS. Although obesity was not found to increase early or late mortality, the higher incidence of postoperative complications highlights the need for careful perioperative planning and monitoring for this patient population. Given the technical challenges associated with obesity, surgical teams should be aware of these factors when making procedural decisions. Importantly, obesity should not be considered a contraindication for MIMVS. Our study has several limitations. First, the retrospective design inherently introduces the possibility of selection bias. Second, although we attempted to adjust for confounding variables, unmeasured factors may have influenced the outcomes, particularly regarding comorbidities and patient characteristics not captured in the dataset. Third, the study was conducted at a single center, which may limit the applicability of these findings to other institutions with lower surgical volumes. Our study presents several strengths that significantly enhance its impact. First, it utilizes a larger sample size compared to other studies, which improves the statistical power and reliability of our findings. Furthermore, the comprehensive long-term follow-up enables a thorough examination of late mortality rates. Additionally, we employed robust statistical methods, including a multivariate regression analysis, to effectively control for confounding variables, facilitating a precise assessment of how obesity influences clinical outcomes and complications.

## 5. Conclusions

In this study, we found that obesity was not associated with increased early or late mortality in patients undergoing MIMVS. However, obese patients experienced higher incidences of postoperative complications, including respiratory insufficiency, arrhythmias, delirium, and wound dehiscence. The complexity of the surgical procedure, reflected in longer operative times and more frequent valve replacements, suggests that obesity presents technical challenges that may influence procedural choices. Nonetheless, obesity itself does not contraindicate MIMVS and should not be viewed as a barrier to offering this minimally invasive approach to obese patients, provided that a well-experienced surgical team and perioperative management are ensured. These findings emphasize the importance of careful perioperative management and suggest that concentrating such procedures in high-volume centers with experienced teams may help optimize outcomes for obese patients. Future studies should focus on examining the long-term effects of obesity on clinical outcomes following MIMVS. Specifically, investigating aspects such as quality of life, functional status, and returning to work after MIMVS will provide valuable insights into the overall impact of obesity on recovery and patient well-being.

## Figures and Tables

**Table 1:** Demographics and preoperative data.

Variables	Non-Obese Group (N = 738)	Obese Group (N = 196)	*p*-Value
Age	64.5 ± 13.9	66.4 ± 11.0	0.08
Elderly cases (over 75 years of age)	196 (26.5%)	50 (25.5%)	0.43
Male Gender	411 (55.6%)	99 (50.5)	0.02 *
Elective operations	492 (66.6%)	122 (62.2%)	0.19
Urgent operations	196 (26.5%)	64 (32.6%)	
Emergency operations	50 (6.7%)	10 (5.1%)	
Peripheral artery disease	53 (7.1%)	17 (8.6%)	0.28
COPD	106 (14.3%)	35 (17.8%)	0.13
Active endocarditis	56 (7.5%)	8 (4%)	0.07
Insulin-dependent diabetes mellitus	18 (2.4%)	18 (9.1%)	0.001 *
Recent myocardial infarction	8 (1.0%)	5 (2.5%)	0.11
Preoperative stroke	58 (7.8%)	16 (8.1%)	0.49
Neurological symptoms	74 (10.0%)	23 (11.7%)	0.51
Pulmonary hypertension	309 (41.8%)	104 (53.0%)	0.03 *
Coronary artery disease	199 (26.9%)	64 (32.6%)	0.12
Smoking history	153 (20.7%)	49 (25%)	0.20
Arterial hypertension	460 (62.3%)	167 (85.2%)	0.001 *
Hyperlipidemia	304 (22.4%)	118 (60.2%)	0.001 *
Atrial fibrillation	313 (42.4%)	111 (56.6%)	0.001 *
Mitral valve stenosis	34 (4.6%)	23 (11.7%)	0.001 *
Mitral valve regurgitation	642 (86.9%)	168 (85.7%)	0.63
Mitral valve prolapse	461(62.4%)	83 (42.3%)	0.001 *
Endocarditis	67 (9.0%)	8 (4.0%)	0.02 *
Anulus dilatation	367 (49.7%)	82 (41.8%)	0.64
Cardiac myxoma	8 (1.0%)	1 (0.5%)	0.87
Mean mitral valve gradient	4 ± 3.5	4.4 ± 2.2	0.25
Maximal mitral valve gradient	8.9 ± 5.1	10.1 ± 5.2	0.007 *

NYHA: New York Heart Association; COPD: chronic obstructive pulmonary disease. * *p*-value significant.

**Table 2:** Procedure characteristics.

Variables	Non-Obese Group (N = 738)	Obese Group (N = 196)	*p*-Value
Biological mitral valve replacement	186 (25.2%)	59 (30.1%)	0.17
Mechanical mitral valve replacement	73 (9.8%)	35 (17.8%)	0.004 *
Mitral valve repair	479 (64.9%)	102 (52.0%)	0.001 *
Plasty with neochordae	343 (46.4%)	61 (31.1%)	0.001
Cleft closure	76 (10.2%)	12 (6.1%)	0.09
Segment resection	38 (5.1%)	4 (2.0%)	0.07
Sliding plasty	8 (1.0%)	1 (0.5%)	0.69
Augmentation	27 (3.6%)	7 (3.5%)	1.0
Maze procedure	133 (18.0%)	40 (20.4%)	0.46
Duration of surgery (minutes)	210.8 ± 54.5	224.1 ± 65.7	0.004 *
Time on CPB (minutes)	78.4 ± 35.3	74.8 ± 39.5	0.21

CPB: cardiopulmonary bypass. * *p*-value significant.

**Table 3:** Postoperative data.

Variables	Non-Obese Group (N = 738)	Obese Group (N = 196)	*p*-Value
In-hospital mortality	14 (1.8%)	6 (3.0%)	0.40 ^§^
30-day mortality	14 (1.8%)	6 (3.0%)	0.40 ^§^
Late mortality	39 (5.3%)	13 (6.6%)	0.48
Duration of therapy with catecholamine	35.5 ± 88.5	47.5 ± 116.9	0.11
Duration of intubation (hours)	27.9 ± 97.0	47.5 ± 116.9	0.06
Erythrocyte transfusion	3.4 ± 5.1	4.1 ± 8.4	0.45
FFP transfusion	1.9 ± 4.7	1.8 ± 6.9	0.89
Platelet transfusion	0.5 ± 1.7	0.6 ± 1.3	0.75
Respiratory insufficiency	35 (4.7%)	25 (12.7%)	0.001 *^§^
Mitral valve re-operation	11 (1.4%)	2 (1.0%)	1.0
Arrhythmia	77 (10.4%)	35 (17.8%)	0.006 *^§^
ECMO/right ventricular failure	23 (3.1%)	7 (3.5%)	0.81
Re-thoracotomy	53 (7.1%)	16 (8.1%)	0.64
Major bleeding	49 (6.6%)	19 (9.6%)	0.16
New onset atrial fibrillation	66 (8.9%)	26 (13.2%)	0.07
Renal failure with new onset dialysis	23 (3.1%)	7 (3.5%)	0.81
Stroke	11 (1.4%)	7 (3.5%)	0.07
Cerebral bleeding	2 (0.3%)	2 (1.0%)	0.19
Seizure	9 (1.2%)	4 (2.0%)	0.48
Delirium	18 (2.4%)	11 (5.6%)	0.03 *^§^
Thromboembolic events	4 (0.5%)	1 (0.5%)	1.0
Wound dehiscence	42 (5.6%)	19 (9.6%)	0.06 *^§^
Sepsis	12 (1.6%)	3 (1.5%)	1.0
Myocardial infarction	2 (0.2%)	0 (0%)	1.0
Pacemaker implantation	38 (5.1%)	16 (8.1%)	0.12
CPR	13 (1.7%)	4 (2.0%)	0.76

CPB: cardiopulmonary bypass; CPR: cardiopulmonary resuscitation; ECMO: extracorporeal membrane oxygenation; FFP: fresh frozen plasma. * *p*-value significant. ^§^ Need to clarify through a multivariate regression analysis.

**Table 4:** Echocardiographic assessments.

Time	Variables	Non-Obese Group (N = 738)	Obese Group (N = 196)	*p*-Value
Preoperative	LVEF	55 ± 16.3	50.7 ± 18.6	0.002 *
	MI II	115 (15.5%)	57 (29.0%)	0.07
	MI III	475 (64.3%)	123 (62.7%)	0.70
	MI IV	71 (9.6%)	10 (5.1%)	0.79
	MS II	8 (1.0%)	8 (4.0%)	0.007 *
	MS III	17 (2.3%)	9 (4.5%)	0.07
Postoperative	LVEF	52 ± 11.2	51.1 ± 13.3	0.37
	MI I	148 (20.0%)	27 (13.7%)	0.07
	MI II	19 (2.5%)	2 (1.0%)	0.27
	MI III	0 (0%)	0 (0%)	-
	MI IV	0 (0%)	0 (0%)	-
	MS II	1 (0.1%)	0 (0%)	1.0
	MS III	1 (0.1%)	0 (0%)	1.0

LVEF: left ventricular ejection fraction; MI: mitral valve insufficiency; MS: mitral valve stenosis; Pre-OP: preoperative; Post-OP: postoperative. * *p*-value significant.

**Table 5:** Multivariate logistic regression analysis.

Intrahospital Mortality				
Variables	OR	Lower–Upper 95% CI		*p*-value
Obesity	3.82	0.15	92.27	0.40
Pulmonary HTN	0.85	0.04	15.92	0.91
Arterial HTN	14.76	0.00	-	0.99
HLP	44.87	0.00	-	0.99
Pre-OP AF	0.77	0.03	15.11	0.86
Mitral valve stenosis	0.00	0.00	-	0.99
Endocarditis	0.00	0.00	-	0.99
Gender	76.63	0.00	-	0.99
IDDM	0.00	0.00	-	0.99
Mitral valve prolapse	1.47	0.06	36.58	0.81
Mechanical MVR	0.00	0.00	-	0.99
Mitral valve repair	0.67	0.02	18.20	0.81
Pre-OP LVEF	1.04	0.90	1.19	0.57
Maximal gradient	1.02	0.67	1.54	0.91
**30-Day Mortality**				
Variables	OR	Lower–Upper 95% CI		*p*-value
Obesity	1.61	0.12	20.36	0.71
Pulmonary HTN	1.53	0.12	19.06	0.73
Arterial HTN	5.00	0.00	-	0.99
HLP	1.42	0.11	17.18	0.78
Pre-OP AF	1.46	0.11	18.50	0.76
Mitral valve stenosis	0.00	0.00	-	0.99
Endocarditis	0.00	0.00	-	0.99
Gender	2.07	0.18	22.92	0.55
IDDM	0.00	0.00	-	0.99
Mitral valve prolapse	0.51	0.03	7.95	0.63
Mechanical MVR	0.00	0.00	-	0.99
Mitral valve repair	0.24	0.01	3.66	0.30
Pre-OP LVEF	1.01	0.93	1.11	0.70
Maximal gradient	0.92	0.72	1.18	0.53
**Wound Dehiscence**				
Variables	OR	Lower–Upper 95% CI		*p*-value
Obesity	1.71	0.885	3.336	0.11
Pulmonary HTN	1.00	0.552	1.838	0.98
Arterial HTN	1.30	0.649	2.613	0.45
HLP	0.73	0.39	1.35	0.32
Pre-OP AF	1.62	0.87	2.99	0.12
Mitral valve stenosis	0.59	0.15	2.32	0.45
Endocarditis	0.87	0.24	3.14	0.83
Gender	0.77	0.43	1.37	0.38
IDDM	2.35	0.80	6.86	0.11
Mitral valve prolapse	0.78	0.38	1.59	0.51
Mechanical MVR	2.34	0.91	6.02	0.07
Mitral valve repair	1.15	0.50	2.63	0.73
Pre-OP LVEF	1.00	0.98	1.02	0.90
Maximal gradient	0.95	0.89	1.02	0.20
**Arrhythmia**				
Variables	OR	Lower–Upper 95% CI		*p*-value
Obesity	1.18	0.68	2.06	0.54
Pulmonary HTN	0.93	0.58	1.51	0.79
Arterial HTN	1.54	0.862	2.75	0.14
HLP	0.88	0.54	1.42	0.60
Pre-OP AF	3.23	1.91	5.48	0.001
Mitral valve stenosis	0.73	0.27	1.97	0.53
Endocarditis	0.00	0.00	-	0.99
Gender	0.96	0.61	1.53	0.89
IDDM	1.50	0.54	4.19	0.43
Mitral valve prolapse	0.84	0.48	1.46	0.54
Mechanical MVR	0.23	0.08	0.64	0.001
Mitral valve repair	0.47	0.26	0.85	0.01
Pre-OP LVEF	1.00	0.99	1.02	0.29
Maximal gradient	1.02	0.97	1.07	0.34
**Delirium**				
Variables	OR	Lower–Upper 95% CI		*p*-value
Obesity	1.03	0.34	3.13	0.95
Pulmonary HTN	1.39	0.53	3.64	0.49
Arterial HTN	0.92	0.30	2.75	0.88
HLP	2.94	0.99	8.73	0.05
Pre-OP AF	2.34	0.83	6.60	0.10
Mitral valve stenosis	0.00	0.00	-	0.99
Endocarditis	1.30	0.15	10.87	0.80
Gender	1.28	0.50	3.26	0.59
IDDM	1.19	0.14	10.07	0.86
Mitral valve prolapse	1.49	0.50	4.43	0.47
Mechanical MVR	0.41	0.04	3.56	0.42
Mitral valve repair	0.80	0.25	2.53	0.71
Pre-OP LVEF	0.99	0.96	1.02	0.80
Maximal gradient	1.03	0.92	1.14	0.59
**Respiratory Insufficiency**				
Variables	OR	Lower–Upper 95% CI		*p*-value
Obesity	0.96	0.44	2.10	0.93
Pulmonary HTN	2.01	0.98	4.09	0.05
Arterial HTN	1.79	0.74	4.31	0.19
HLP	1.89	0.91	3.92	0.08
Pre-OP AF	1.37	0.68	2.77	0.37
Mitral valve stenosis	0.54	0.11	2.57	0.44
Endocarditis	0.83	0.17	3.93	0.81
Gender	0.85	0.43	1.68	0.64
IDDM	0.79	0.17	3.70	0.77
Mitral valve prolapse	0.94	0.42	2.07	0.88
Mechanical MVR	0.96	0.34	2.73	0.95
Mitral valve repair	0.75	0.32	1.74	0.51
Pre-OP LVEF	0.97	0.96	0.99	0.01
Maximal gradient	1.03	0.96	1.10	0.32

AF, atrial fibrillation; CI, confidence interval; HLP, hyperlipidemia; HTN, hypertension; IDDM, insulin-dependent diabetes mellitus; LVEF, left ventricular ejection fraction; MVR, mitral valve replacement; OR, odds ratio; Pre-OP, preoperative; Post-OP, postoperative.

## Data Availability

Data are contained within the article.
